# Resonance-Based Sparse Signal Decomposition and Its Application in Mechanical Fault Diagnosis: A Review

**DOI:** 10.3390/s17061279

**Published:** 2017-06-03

**Authors:** Wentao Huang, Hongjian Sun, Weijie Wang

**Affiliations:** School of Mechatronics Engineering, Harbin Institute of Technology, No. 92 Xidazhi Street, Harbin 150001, China; hongjiansunny@163.com (H.S.); greatwang@hit.edu.cn (W.W.)

**Keywords:** resonance-based sparse signal decomposition, signal processing, mechanical fault diagnosis, feature extraction

## Abstract

Mechanical equipment is the heart of industry. For this reason, mechanical fault diagnosis has drawn considerable attention. In terms of the rich information hidden in fault vibration signals, the processing and analysis techniques of vibration signals have become a crucial research issue in the field of mechanical fault diagnosis. Based on the theory of sparse decomposition, Selesnick proposed a novel nonlinear signal processing method: resonance-based sparse signal decomposition (RSSD). Since being put forward, RSSD has become widely recognized, and many RSSD-based methods have been developed to guide mechanical fault diagnosis. This paper attempts to summarize and review the theoretical developments and application advances of RSSD in mechanical fault diagnosis, and to provide a more comprehensive reference for those interested in RSSD and mechanical fault diagnosis. Followed by a brief introduction of RSSD’s theoretical foundation, based on different optimization directions, applications of RSSD in mechanical fault diagnosis are categorized into five aspects: original RSSD, parameter optimized RSSD, subband optimized RSSD, integrated optimized RSSD, and RSSD combined with other methods. On this basis, outstanding issues in current RSSD study are also pointed out, as well as corresponding instructional solutions. We hope this review will provide an insightful reference for researchers and readers who are interested in RSSD and mechanical fault diagnosis.

## 1. Introduction

With the rapid development of modern industry, mechanical equipment, the industrial heart, is developing towards the direction of large scale, high speed, high accuracy and system integration. Due to harsh working conditions, the faults of mechanical components may strike randomly, and more frequently in their later life. Even the fault of a single component is likely to result in the shutdown of an entire piece of mechanical equipment, especially when considering the chain effects. Thus, mechanical faults may cause huge economic costs and even catastrophic casualties [1,2]. For instance, in 2006, damage of the propulsion system was induced by a gearbox fault in the ship “Zhouying 4”, causing enormous pecuniary loss for the Chinese government [3]. Advanced fault diagnosis technology can not only detect mechanical faults as early as possible, before fatalities, but also fundamentally solve the problem of inadequate and excessive maintenance, which will be of great benefit to the safe operation of mechanical equipment. As a result, great attention has been paid to fault diagnosis technology.

**Figure 1:**
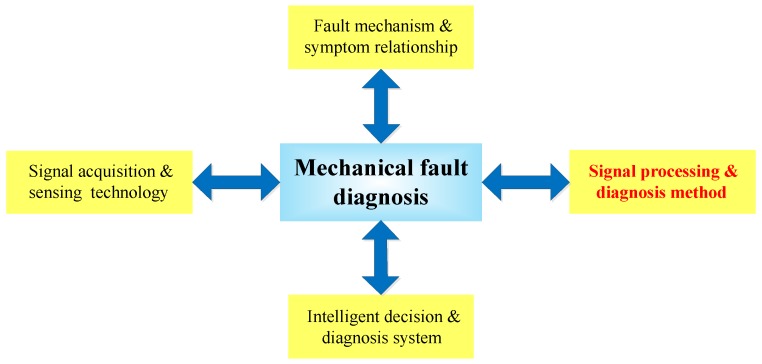
Research directions of mechanical fault diagnosis.

Mechanical fault diagnosis is a comprehensive and interdisciplinary study, since it combines monitoring, diagnosis, and prognostics. Its major research directions include signal acquisition and sensing technologies, fault mechanisms and symptom relationships, signal processing and diagnostic methods, and intelligent decision and diagnosis systems [4], as shown in Figure 1. Among these, signal processing and diagnosis methods are researched most extensively. It is acknowledged that vibration analysis is the most effective tool for mechanical fault diagnosis [5,6]. Aiming at extracting information on fault features and subsequently recognizing mechanical fault types, a wealth of signal processing methods are continuously put forward and applied to fault vibration signal analysis. To date, fault feature extraction techniques based on signal processing include short-time Fourier transformation (STFT) [7], wavelet transformation (WT) [8], empirical mode decomposition (EMD) [2], resonance demodulation [9], and morphological operators [10], etc. Although these techniques have found wide application in mechanical fault diagnosis, there are still some problems remaining to be settled. For example, the basis function of wavelet transformation cannot be altered once selected; mode mixing problems are common in EMD; and center frequency and bandwidth are difficult to determine for resonance demodulation. Due to lacking effective solutions to the above problems, misdiagnosis and even missed diagnosis emerge in many cases, which become the biggest constraints in theory and engineering applications. In short, the feature extraction method of mechanical fault vibration signal still needs to be further perfected.

Resonance-based sparse signal decomposition is a novel nonlinear signal processing method that was proposed by Selesnick in 2011 [11]. The method takes resonance as an intrinsic property of a signal and evaluates resonance degree with a quality factor (defined as the ratio of the center frequency and frequency bandwidth, denoted *Q*). A signal with stronger resonance signifies that it possesses a higher *Q*, oscillates more times in a time domain, and its frequency band is distributed more intensively; and vice versa. Unlike frequency band based signal analysis methods, such as WT and EMD, RSSD realizes the nonlinear separation of each component according to its oscillation property (resonance) through morphological component analysis (MCA) [12], and obtains the sparsest representation of each resonance component. So far, RSSD has been widely applied in speech signals [13,14], biological signals [15,16], power systems [17,18,19], and other fields [20,21,22,23], and the relevant literature has mushroomed over the past few years.

The fault vibration signals collected from mechanical systems—mainly bearings, gearboxes, and rotor systems—usually exhibit a superposition of periodic fault-induced vibration responses and random noise. Indeed, periodic signal components and background noise can correlate well with the high- and low-resonance components in RSSD, especially from the viewpoint of resonance. Moreover, since RSSD breaks through the limitations of traditional techniques where processing signals are based on frequency band, it is more suitable to process mechanical fault vibration signals with obvious nonlinear and non-stationary characteristics. Hence, related research in the mechanical fault diagnosis field is increasingly heated. Unfortunately, our literature survey reveals that a review on the applications of RSSD in mechanical fault diagnosis has not been published.

To fill up the review gap, this paper attempts to summarize the theoretical developments and applications of RSSD in mechanical fault diagnosis. For the purpose of centralizing, categorizing, and analyzing these individual studies, this paper provides a more comprehensive reference and promotes the appearance of more advanced research about RSSD, which can make a great contribution to mechanical fault diagnosis. The rest of this review is organized as follows. In Section 2, a brief introduction about signal resonance and two construction methods of wavelet bases are presented. Section 3 presents an implementation algorithm for RSSD, illustrates its performance, and investigates its parameters’ influences on decomposition results. Section 4 reviews recent application advances of RSSD in mechanical fault diagnosis based on different optimization directions. In Section 5, these methodologies and applications are synthesized in a flow chart and table. Outstanding issues and solutions of RSSD in mechanical fault diagnosis are discussed in Section 6. Finally, concluding remarks are provided in Section 7.

## 2. Theoretical Foundation of Wavelet Bases

Due to the differences in signal resonance, RSSD is able to decompose complex signals into high-resonance components comprising sustained oscillation signals, low-resonance components consisting of transient impulse signals, and residual components. Noteworthy, the core superiority of RSSD lies in the construction of wavelet bases. Thus, after a brief explanation of signal resonance, this section will concentrate on the construction methods of wavelet bases, including rational-dilation wavelet transformation (RADWT) [24,25] and tunable *Q*-factor wavelet transformation (TQWT) [26].

### 2.1. Signal Resonance

The signal resonance property can be described with the *Q*-factor, which is defined as the ratio of the center frequency *f_c_* and bandwidth *BW* in frequency domain, as expressed in Equation (1):

(1) $$Q=\frac{f_{c}}{BW}$$

**Figure 2:**
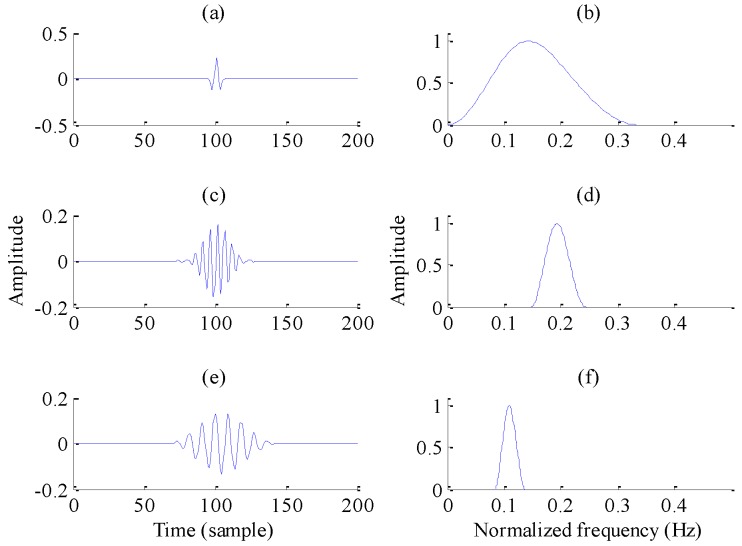
Signals with different *Q*-factors: (**a**,**b**) *Q* = 1; (**c**,**d**) *Q* = 4; (**e**,**f**) *Q* = 4.

Figure 2 explains the resonance property in more detail. Note that the left section of this figure shows the time-domain waveforms, with their corresponding frequency spectra on the right. For Figure 2a,b, the time-domain signal resembles an impulse signal and exhibits no sustained oscillatory behavior; thus it is defined as the low-resonance component. For the signals in Figure 2c–f, whose *Q*-factors are equal to 4, their oscillations are about eight times in time domain and sustained oscillatory behaviors are observed, so they are defined as the high-resonance components. Comparing Figure 2b,d or Figure 2f, it is evident that a higher-*Q* signal has a more concentrated frequency distribution for a fixed frequency than a low-*Q* signal. Moreover, the signals in Figure 2c,e are likely able to convert to the other one through compressed or stretched transformations in their time domains. This time-scaling transformation doesn’t change their resonance degrees, which explains why they have *Q*-factors. Also, their frequency distributions are nonoverlapping, in accordance with the fact that the resonance property is independent of time scale and frequency.

Based on signal resonance, Selesnick successively put forward RADWT and TQWT to construct the wavelet bases used in RSSD, and they are both overcomplete discrete wavelet transformations.

### 2.2. Basic Theory of RADWT

Dyadic wavelet transformation is a common constant-*Q* transformation, but a low *Q*-factor limits the application in some conditions where a high frequency resolution is strictly requested [25]. To overcome this shortcoming, Selesnick developed RADWT in 2009 and successfully applied it to RSSD in subsequent years [11].

**Figure 3:**
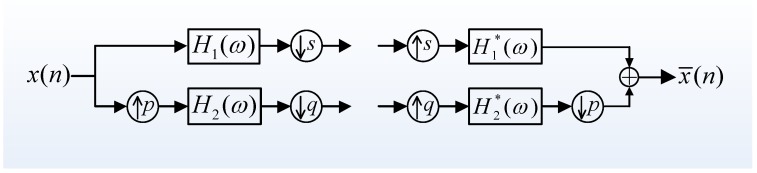
Two-channel filter banks of rational-dilation wavelet transformation (RADWT).

On a theoretical level, like the tree-structure filter bank for Dyadic wavelet transform, RADWT is also implemented by using the two-channel filter banks illustrated in Figure 3, except for the difference that only one sampling process exists in the high-pass filter.

Tunable parameters in RADWT include *p*, *q*, and *s*, and the dilation factor *d* is defined as *p*/*q*, wherein co-prime numbers *p*, *q* should satisfy *p* < *q* and *p*/*q* + 1/*s* > 1. In effect, the *Q*-factor is adjusted with the combined action of *p*, *q*, and *s*, as in Equation (2):

(2) $$Q=\sqrt{d}\frac{1}{1-d}$$

It must be mentioned that this formula for the *Q*-factor is feasible only when the following equation is satisfied:

(3) $${\left(\frac{p}{q}\right)}^{2}<1-\frac{1}{s}$$

Without such a guarantee, it will be extremely difficult to find an accurate formula for calculating *Q*-factors. Equation (3) enables the *Q*-factor to increase generally with increasing dilation factor *d*. Particularly when the value of *d* gradually approaches zero, the *Q*-factor will increase rapidly according to Equation (2), and the RADWT wavelet will have a more concentrated frequency, as well as a finer frequency resolution.

On the other hand, *s* with a value of 1 will lead to transient wavelet features in time domain, which usually indicates the low-resonance components. A value larger than 1 for *s* allows the constructed wavelets to correlate well with high-resonance components, since the subband wavelets exhibit sustained oscillations in this case. Comparatively speaking, wavelet oscillatory characteristics are more sensitive to *s* than to *d*, and increasing *s* can effectively strengthen the time-domain oscillation and frequency concentration. A sufficiently large *s* will avoid the “ringing” phenomenon, but result in a “brick wall” frequency distribution [25].

Assuming Equation (3) is satisfied, and given input signal sampling frequency *f_s_*, center frequency *f_c_* and bandwidth *BW* of the level-*j* subband wavelet constructed by RADWT can be calculated:

(4) $$f_{c}=d^{j-3/2}(1-\frac{1}{s}+d)f_{s}$$

(5) $$BW=2d^{j-2}(1-\frac{1}{s}+d)(1-d)\pi$$

**Figure 4:**
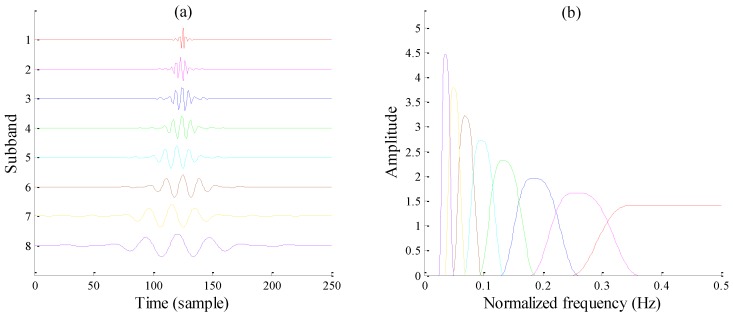
A typical RADWT wavelet base with *p* = 18, *q* = 25, *s* = 2. (**a**) Time-domain waveforms; (**b**) Frequency responses.

Figure 4 displays a typical case for RADWT subband bases and their frequency responses. Note that values of *p* and *q* are specially chosen to make the *Q*-factor close to 3, consistent with the following TQWT. It can be seen that all subbands show obvious oscillations in the time domain, and the frequency distributions of first several subbands both have flat tops, which appear only when Equation (3) comes into existence. In the meantime, the peak amplitudes of each subband vary from each other and show an overall growth trend.

Consequently, the proposal of RADWT greatly broadens the scope of *Q*-factors and improves the flexibility of selecting *Q*-factors. Nevertheless, *p* = 18 and *q* = 25 can only yield a value approximating 3, rather than a precise *Q*. If pursuing a more accurate *Q*, larger values of *p* and *q* are unavoidable because they are co-prime. This will result in the length increase of the under-decomposed signal, which blocks RADWT’s applications to more general occasions. In terms of Equation (3), an accurate calculation for the *Q*-factor is also unreachable in some cases, which makes it very difficult to select an exact *Q*-factor assisted with a priori knowledge. More unfortunately, without calculation formulas for *f_c_* and *BW* like those given in Equations (4) and (5), subsequent analysis and optimization on subbands are enormously challenging, lacking theoretical foundation. Taking these restrictions into consideration, a more advantages transformation needs to be developed.

### 2.3. Basic Theory of TQWT

**Figure 5:**
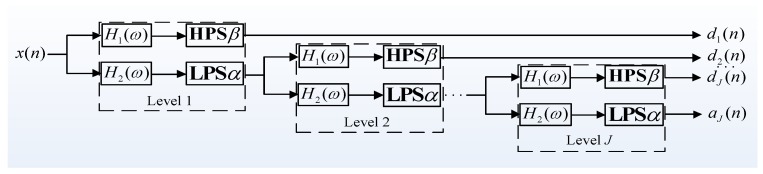
The analysis filter banks of tunable *Q*-factor wavelet transformation (TQWT).

**Figure 6:**
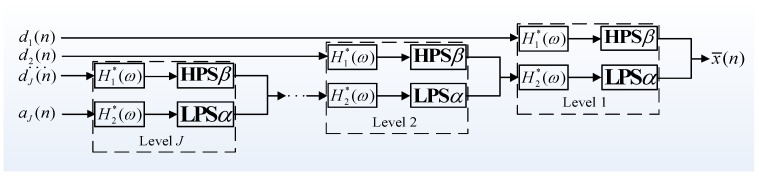
The synthesis filter banks of TQWT.

As a significant breakthrough in constructing wavelet bases, TQWT overcomes the length limitation of the input signal excellently, and has a more extensive application prospects than RADWT. Via directly specifying the *Q*-factor, redundancy factor *r*, and decomposition level *J*, wavelet bases are designed. Obviously, TQWT has more flexibility than RADWT; therefore, it is not only favored by RSSD researchers in the field of mechanical fault diagnosis, but also in most signal analysis fields [27]. Briefly, the implementation of TQWT also depends on iterative analysis and synthesis filter banks, as shown in Figure 5 and Figure 6, respectively.

In Figure 5 and Figure 6, **HPS** and **LPS** represent high- and low-pass scaling, and β and α are the corresponding scaling parameters, which satisfy 0<α<1, 0<β<1, α+β>1. $H_{1}(\omega )$ and $H_{2}(\omega )$ are the high- and low-pass frequency responses, respectively.

The parameters in TQWT consist of the *Q*-factor, redundancy factor *r*, and decomposition level *J*, whose concrete descriptions are listed in Table 1.

The *Q*-factor describes the degree of signal resonance, and needs to be selected according to the characteristics of practical signals. Redundancy factor *r* controls the overlapping rate between the frequency responses of adjacent wavelets. With a fixed *Q*-factor, an increase in redundancy factor *r* will lead to a higher overlapping rate. Note that redundancy factor *r* should be strictly greater than 1; a value greater than 3 is often recommended for the perfect reconstruction and sparsity. Once the *Q*-factor and redundancy factor *r* are determined, the scaling parameters β and α can be obtained through the following equation:

(6) β=2/(Q+1), α=1−β/r

The decomposition level, *J*, adjusts the frequency coverage of the wavelets, with a higher *J* enabling the wavelets to cover a wider frequency range and approach 0 Hz. However, excessive decomposition (an overlarge *J*) will lead to poor computational efficiency. Specifically, TQWT decomposes an *n*-point discrete-time signal into *J*-level subbands, including the detail coefficients and approximated coefficients. The maximum number of levels is set according to the following equation ([·] represents the “round down to the nearest whole unit”):

(7) $$J_{\max}=\left[\frac{\log(\beta *n/8)}{\log(1/\alpha )}\right]$$

The center frequency *f_c_* and bandwidth *BW* of the obtained level-*j* subband can be approximated:

(8) $$f_{c}=\alpha^{j}\frac{2-\beta }{4\alpha }f_{s}$$

(9) $$BW=\frac{1}{2}\beta \alpha^{j-1}\pi$$

**Figure 7:**
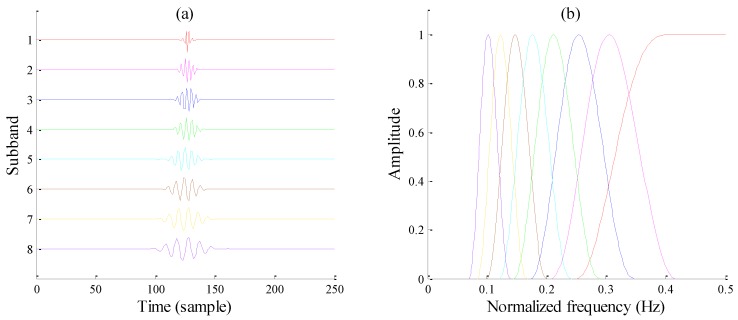
A typical TQWT wavelet bases with *Q* = 3, *r* = 3, *J* = 8. (**a**) Time-domain waveforms; (**b**) Frequency responses.

Analyzing Equations (6), (8), and (9) jointly, it can be deduced that the tunable *Q*-factor wavelet bases can be entirely constructed with the determined *Q*-factor, redundancy factor *r*, and decomposition level *J*. Figure 7 plots a typical wavelet basis, with *Q* = 3, *r* = 3, and *J* = 8. As the decomposition levels increase, the corresponding center frequency and bandwidth are both lessened, while the *Q*-factor stays unchanged.

By comparing the TQWT and RADWT subbands, it can be observed that the frequency responses of the TQWT subbands no longer have flat tops except for the first level subband, and their frequency peaks each maintain a constant value. Instead of carefully choosing RADWT parameters *p*, *q*, and *s*, an explicit *Q*-factor indicating resonance behavior is much more convenient to obtain with TQWT, especially when signal oscillatory behavior is known in advance. Furthermore, formulas for calculating TQWT subband center frequency *f_c_* and bandwidth *BW* greatly promote deeper developments for extracting information of interest from subband signals, which makes TQWT more popular for many conditions.

## 3. Resonance-Based Sparse Signal Decomposition

### 3.1. RSSD Implementation Algorithm

The objective of RSSD is to separate the different resonance components of a given signal and realize the sparsest representation of each resonance component. In particular, for a given signal **x** = **x**_1_ + **x**_2_, via RSSD the signal is decomposed into the high-resonance component **x**_1_, low-resonance component **x**_2_, and the residual, assuming that **x**_1_ and **x**_2_ can be sparsely represented in bases **S**_1_, **S**_2_ (constructed by TQWT with high and low *Q*-factors), respectively. Hence, a suitable optimization problem for estimating coefficient matrixes **W_1_** and **W_2_** under **S**_1_ and **S**_2_ is:

(10) $$W_{1},W_{2}=\underset{W_{1},W_{2}}{\arg\min}\left\{{\left\|x-S_{1}W_{1}-S_{2}W_{2}\right\|}_{2}^{2}+\lambda_{1}{\left\|W_{1}\right\|}_{1}+\lambda_{2}{\left\|W_{2}\right\|}_{1}\right\}$$

where $\lambda_{1},\lambda_{2}$ are the corresponding weight coefficients. It is worth mentioning that relative values of $\lambda_{1},\lambda_{2}$ determine the energy distributions of these two resonance components. With a fixed $\lambda_{1}$, increasing $\lambda_{2}$ will increase the energy of x_1_ and decrease the energy of x_2_, and vice versa. Increasing both $\lambda_{1},\lambda_{2}$ will increase the energy of the residual and decrease that of the resonance components.

Although the optimization problem in Equation (10) is convex, a large number of variables and the non-differentiability of the *l*_1_-norm make it difficult to solve [11]. Thankfully, the Split Augmented Lagrangian Shrinkage Algorithm (SALSA) [28,29] has been proven as a powerful tool to handle the optimization problem. With SALSA, RSSD iteratively updates coefficient matrixes **W**_1_, **W**_2_ to achieve the minimization. At the end of all iterations, optimal coefficient matrixes $W_{1}^{*},W_{2}^{*}$ are gained, and the resonance components also reach a relatively considerable state for fault feature extraction, although they are not always sparse in terms of random noises. At this time, estimates of the high- and low-resonance components are provided based on MCA:

(11) $${\bar{x}}_{1}=S_{1}W_{1}^{*},\;{\bar{x}}_{2}=S_{2}W_{2}^{*}$$

More details about RSSD can be found in Reference [11]. Summarizing the contents of Section 2.3 and Section 3.1, the concrete steps of RSSD are as follows:

Step 1 Input a signal x for decomposition;
Step 2 Assisted with a priori information, select suitable *Q*-factors *Q*_1_, and *Q*_2,_ redundancy factors *r*_1_, and *r*_2_, and decomposition levels *J*_1_, and *J*_2_ to construct corresponding wavelet bases **S**_1_, **S**_2_ via TQWT;
Step 3 According to the observation signal noise level, determine suitable weight coefficients $\lambda_{1},\lambda_{2}$ and establish Equation (10);
Step 4 Solve the optimization problem with SALSA and obtain the optimal coefficient matrixes $W_{1}^{*},W_{2}^{*}$;
Step 5 Utilize $W_{1}^{*},W_{2}^{*}$ to represent the estimates of high- and low-resonance components with ${\bar{x}}_{1}=S_{1}W_{1}^{*},\;{\bar{x}}_{2}=S_{2}W_{2}^{*}$.

To clearly demonstrate the superiority of RSSD in decomposing different resonance signals, a performance illustration is presented in the next subsection.

### 3.2. Illustration of RSSD Performance

**Figure 8:**
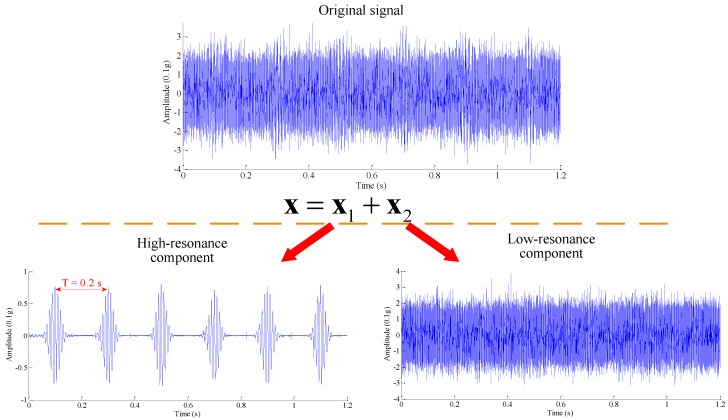
Illustration of resonance-based sparse signal decomposition (RSSD) performance.

In this subsection, to verify the effectiveness of RSSD in processing mechanical vibration signals, a typical gear fault vibration signal is constructed as follows (the Morlet function):

(12) $$y(t)=\sum_{k=0}^{5}e^{\frac{\zeta }{\sqrt{1-\zeta^{2}}}{\left[2\pi f(t-kT-\tau )\right]}^{2}}\cos\left[2\pi f(t-kT-\tau )\right]$$

where damping ratio ζ=0.01, frequency *f* = 100 Hz, time shift τ=0.1 s, and cyclic period *T* = 0.2 s. Meanwhile, gaussian noise with a signal-noise ratio 4dB is also added, which makes the simulation signal closer to the practical one. In this example, the gear fault-induced signal components, in the form of the Morlet function, belong to the high-resonance component, and noises should fall into the low-resonance category for their non-oscillatory behaviors. Set decomposition parameters to *Q*_1_ = 4, *r*_1_ = 5, *J*_1_ = 105, $\lambda_{1}=0.3$, *Q*_2_ = 1, *r*_2_ = 5, *J*_2_ = 10, and $\lambda_{2}=0.1$, and employ standard RSSD to process the composite signal. The original signal, and obtained high- and low-resonance components, are displayed in Figure 8.

It can be seen that fault-related waveforms are almost buried due to the presence of strong background noises, and thus no obvious fault information can be found easily in original signal. Moreover, the oscillatory waveforms and noises overlap, either in time domain or in frequency domain, which will invalidate traditional frequency band based methods. In RSSD space, the original signal can be decomposed into two parts: (1) gear fault-induced sustained waveforms; (2) noises with non-oscillatory features. With inspection, the results conform the theoretical analysis: the high-resonance component mainly consists of sustained oscillatory Morlet waveforms, while the transient characteristic of the low-resonance component is apparent. Meanwhile, the period 0.2 s for the high-resonance component further indicates an obvious gear fault.

In addition to validating RSSD’s effectiveness, we will also analyze the parameters’ influences on decomposition results, especially on the high- and low-resonance waveforms.

### 3.3. Effects of RSSD Parameters

Since the redundancy factor *r* and decomposition level *J* only affect the overlapping rate and frequency coverage, they can cooperate with each other to achieve the frequency ranges requested in RSSD. As such, only the effects of the *Q*-factors and weight coefficients are further investigated.

#### 3.3.1. Effects of *Q*-Factors

**Figure 9:**
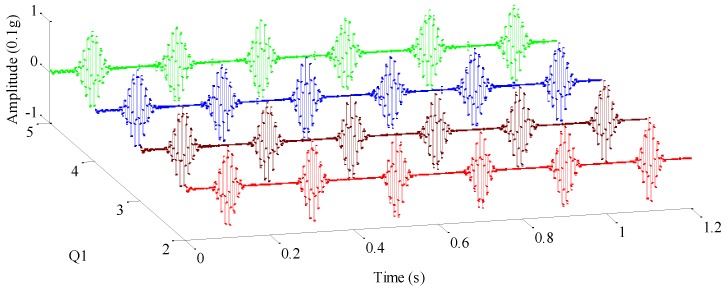
High-resonance components in cases where *Q*_1_ = 2, 3, 4, 5.

Generally, the low *Q*-factor is set as 1, which satisfies the requirement of representing transient noises in a complex signal. However, a high *Q*-factor needs to be selected assisted with a priori information. Therefore, we will further analyze the mechanical vibration signal simulated above through altering the high *Q*-factor *Q*_1_ and obtaining the high-resonance components for a different *Q*_1_, as shown in Figure 9. For the sake of clarity, Figure 9 only plots high-resonance components when *Q*_1_ is equal to 2, 3, 4 and 5, respectively. Noteworthy, the high-resonance component in blue corresponds to the illustration of RSSD performance shown in Section 3.2, with identical decomposition parameters. As seen in Figure 9, high-resonance waveforms seem not to exhibit distinct differences though *Q*_1_ varies from 2 to 5.

To give a quantitative evaluation to this indistinctive variation, the degree of similarity between resonance components and the original signal is utilized with the correlation coefficient equation [30,31]

(13) $$\rho_{i}=\frac{\left|\sum_{k=1}^{N}\left(x_{i}(k)-mean({\bar{x}}_{1}))(x(k)-mean(\bar{x})\right)\right|}{\sqrt{\sum_{k=1}^{N}{\left(x_{i}(k)-mean({\bar{x}}_{1})\right)}^{2}\sum_{k=1}^{N}{\left(x(k)-mean(\bar{x})\right)}^{2}}}\;i=1,2$$

where $x_{i}(k)$ is the element in high- and low-resonance components respectively, x(k) is the element in the original signal, and *mean* represents the averaging operation. Thus, we obtain the correlation coefficients $\rho_{1},\rho_{2}$ between the resonance components and the original signal, respectively, as listed in Table 2. From Table 2, it is easy to discover that correlation coefficients change slightly when *Q*_1_ varies from 2 to 9 for both the high-resonance component and low-resonance component. The maximum variations of high- and low-resonance components are only 0.08% and 0.44%, respectively. It is the tiny variations that explain why the high-resonance waveforms don’t show obvious differences. Meanwhile, Table 2 also indicates that the selection of a high *Q*-factor has some effects on the decomposition results, or rather an exacter description—‘limited effects’.

#### 3.3.2. Effects of Weight Coefficients

**Figure 10:**
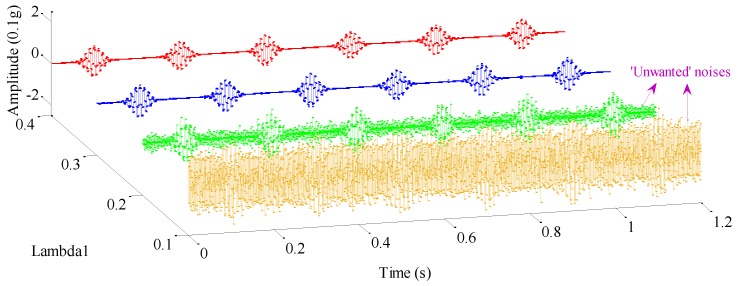
High-resonance components in cases where $\lambda_{1}=0.1,0.2,0.3,0.4$.

**Figure 11:**
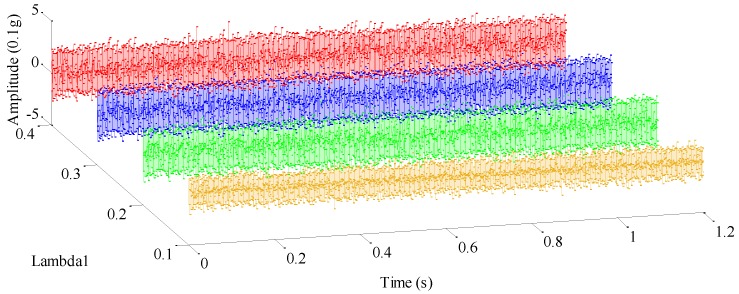
Low-resonance components in cases where $\lambda_{1}=0.1,0.2,0.3,0.4$.

As analyzed, weight coefficients $\lambda_{1},\lambda_{2}$ control the energy distribution between each resonance component. To highlight the significant influence caused by variations of the weight coefficients, we consider the condition that only $\lambda_{1}$ varies. Figure 10 and Figure 11 plot the high- and low-resonance components when $\lambda_{1}$ is equal to 0.1, 0.2, 0.3, and 0.4 respectively, whose correlation coefficients are provided in Table 3. As is easily seen in Figure 10 and Figure 11, as the weight coefficient $\lambda_{1}$ changes, both high- and low-resonance components undergo considerable waveform changes, especially the high-resonance component.

In detail, when $\lambda_{1}=0.4,0.3$, the high- and low-resonance components can be clearly separated. Though amplitudes of both high-resonance components decay a little bit, their periodical oscillatory features are fairly dominant, which indicates a satisfactory decomposition result for mechanical fault diagnosis.

When $\lambda_{1}=0.2$, amplitude of the high-resonance waveform increases significantly, but slight noise springs up as well, compared with the condition when $\lambda_{1}$ is 0.4 or 0.3, as described above. Consequently, the sparsity of high-resonance components cannot be guaranteed due to the emergence of unwanted noise.

Furthermore, the case where $\lambda_{1}$ is equal to 0.1 is discussed. At this time, sustained oscillatory characteristics can hardly be distinguished in the high-resonance component, since noise is rather abundant, which will make few contributions to mechanical fault feature extraction. Reflecting on the low-resonance components in the above cases, with an increasing $\lambda_{1}$, the most evident variation lies that their amplitudes increase gradually. Considering that there is less fault information embedded in low-resonance components, high-resonance components are more suitable for the detection of mechanical fault features.

A summary of the information in Table 3 shows that maximum correlation coefficient variations reach up to the surprising levels of 61.5% and 15.69% for the high- and low-resonance components, respectively. With a careful observation, the correlation coefficient of the high-resonance component undergoes a sharp variation when $\lambda_{1}$ steps from 0.1 to 0.2, the primary cause of which is the rich presence of noise. In short, such a quantitative result demonstrates that weight coefficients have a remarkable effect on the decomposition results of RSSD, and they must be treated with caution.

In spite of the outstanding performance of RSSD in processing nonlinear and non-stationary signals, whether RSSD can be conducted successfully relies not only on signal intrinsic characteristics, but also on the selection of ideal parameters to a great extent. By contrast, weight coefficients have a more significant and even decisive effect on decomposition results than *Q*-factors. As a result, to guarantee applied success, the optimized selection of *Q*-factors and weight coefficients is the most crucial issue for developing more advanced RSSD and promoting wider applications.

## 4. Applications of RSSD in Mechanical Fault Diagnosis

Due to the rich fault-related information buried in noisy mechanical fault signals, fault diagnosis technique based on signal processing can extract sensitive features and identify fault types. RSSD separates complex signals based on a new perspective, resonance, overcomes the limitation of traditional frequency band based methods, and can reveal fault features from original mechanical vibration signals more effectively. This huge advantage allows RSSD to be extensively applied to the fault diagnosis of crucial industrial components, that is, bearings, gearboxes and rotors. To present an organized review, this section will provide a survey of the applications of RSSD in mechanical fault diagnosis based on different optimization directions, including original RSSD, parameter optimized RSSD, subband optimized RSSD, integrated optimized RSSD, and RSSD combined with other methods.

### 4.1. Original RSSD in Mechanical Fault Diagnosis

**Figure 12:**
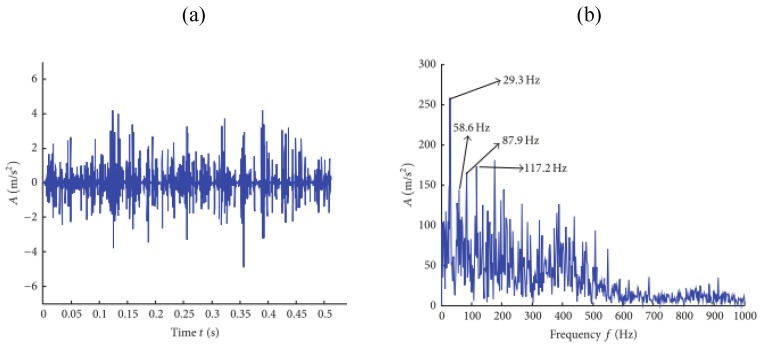
Low-resonance component of gearbox fault vibration signal. (**a**) Time-domain waveforms; (**b**) Demodulation spectrum [38].

Up to now, the original RSSD proposed by Selesnick in 2011 has been extensively applied in the field of mechanical fault diagnosis. This subsection will review the publications in which only original RSSD was used, without combining other techniques. In 2012, Yu et al. [32] pioneered the introduction of RSSD into the field of mechanical fault diagnosis and proposed an envelope demodulation method based on RSSD. This method separated transient impacts containing bearing fault information into their low-resonance components, and executed envelope demodulation analysis on the low-resonance components to detect the rolling bearing’s inner and outer fault features successfully. Afterwards, a similar method was also employed for the fault diagnosis of gears [33]. Xiang et al. [34,35] and Huang et al. [36,37] performed RSSD to decompose rolling bearing fault vibration signals into high- and low-resonance components, effectively reduced heavy background noise, and extracted weak rolling bearing fault information quickly and accurately. In consideration of the frequency overlap between gearbox fault vibration signal components, RSSD was utilized [38,39]. The obtained low-resonance component of the engineering gearbox fault signal, as well as its envelope spectrum, is illustrated in Figure 12. Since the gear fault feature frequency was 30.1 Hz, the peaks at 29.3 Hz and harmonics were cogent enough to indicate a gear fault. For the sake of safety in production, the factory checked and confirmed the broken gear teeth a few weeks later.

Zhang et al. [40] introduced an energy operator demodulating analysis method based on RSSD and detected compound fault features of a gearbox. To segment the impacts from vibration signals in rotor systems, Chen et al. [41,42] performed RSSD and diagnosed early rub-impact fault with the reassigned wavelet scalogram. In 2014, Wang et al. [43] used TQWT to construct high- and low-*Q*-factor wavelet bases to successfully separate the periodic component with rotor rotation rate from transient component induced by rub-impact fault. Accurate fault identification from the low-resonance component verified RSSD’s validity. In addition, a condition assessment system for an automatic tool changer in CNC machining centers was established by Chen [44]. This monitoring system, based on RSSD, could not only detect the transient component caused by a globoidal indexing cam fault, but also locate the fault’s exact position.

It is interesting that a group of scholars represented by Yu, Cui, and Chen thought the vibration responses induced by mechanical fault impact exhibited transient characteristics, which should fall under the category of the low-resonance component, while the high-resonance component sparsely represented the high-frequency interferences. In sharp contrast, Huang’s team considered the fault impact as a damped oscillation response, whose amplitude diminished continuously but still exhibited sustained oscillation behaviors while the low-resonance component corresponded to random interference in the original signal. Thus Huang preferred to discover fault information from the high-resonance component rather than from the low-resonance component. In fact, all fault-related information cannot be completely decomposed into a single resonance component, and energy leakage was inevitable due to their inherent mutual coherence. Yu et al. made use of the low-resonance component to seize the instantaneous peak induced by mechanical fault impact, while Huang utilized the high-resonance component to catch the characteristics of damped oscillations. These two detection methods were both reasonable and feasible in theory. Moreover, the fact that they all extracted the mechanical fault features in their own way confirms the energy leakage phenomenon. In short, mechanical fault-induced feature information will simultaneously lie in the high-resonance component, the low-resonance component, and even the residual.

The original RSSD has been proven effective for feature extraction, and has a promising application in mechanical fault diagnosis. However, in consideration of the RSSD method and its application, some key points remain to be further investigated:

(1) Construction of wavelet bases;
(2) Optimized selection of numerous decomposition parameters;
(3) Reconstruction of subband signals;
(4) Sparse decomposition of multiple resonances.

At the present time, further research on RSSD mainly focuses on two aspects, the optimized selection of parameters (Point 2), and the reconstruction of subband signals (Point 3).

### 4.2. Parameter Optimized RSSD in Mechanical Fault Diagnosis

The analysis in Section 3.2 explains that parameters (*Q*-factors and weight coefficients) appreciably influence decomposition results, and that the effects of weight coefficients are much more predominant and crucial. Therefore, abundant literatures have paid much attention to selecting optimal decomposition parameters, seeking more effective application for mechanical fault diagnosis. Dai and Cui [45,46] comprehensively studied the effects of *Q*-factors, redundancy factors, and weight coefficients, and emphasized weight coefficients’ effects in RSSD. Some efficient suggestions were provided by Huang for selecting the decomposition level *J* in conjunction with a specific diagnosis objects: rolling bearings [47]. Coincidentally, both stressed the suitable selection of weight coefficients for feature extraction of early weak faults in rolling bearings. The conclusions from these studies are completely consistent with our analysis in Section 3.2. Regretfully, no index or indicator was adopted in these studies to evaluate the influences. Consequently, Cai et al. [48] offered a new method to select weight coefficients. In this method, three indexes were set up to assess the decomposition results, and weight coefficients corresponding to better results were adopted. Then RSSD with optimized parameters was used to successfully detect the compound fault features of a gearbox. To overcome the large subjective randomness when selecting RSSD parameters, an optimized RSSD based on the genetic algorithm (GA), and realizing the adaptive decomposition of high- and low-resonance components, was proposed in Reference [49] by concurrently selecting and optimizing the elements of coefficient matrixes. This method minimized information leakage in the process of signal decomposition and effectively extracted compound fault characteristics of rolling bearings. On this basis, Huang et al. [50] made full use of the global optimization ability of GA, and optimized *Q*-factors further. RSSD with the optimal *Q*-factors was used successfully to diagnose the composite faults of the planetary gear and bearing in a planetary gearbox. Similar work was also performed in Reference [51], though one difference that must be mentioned was that Li took the kurtosis of low-resonance component as the objective function used in GA, instead of a function like Equation (10), utilized in Huang’s research.

**Figure 13:**
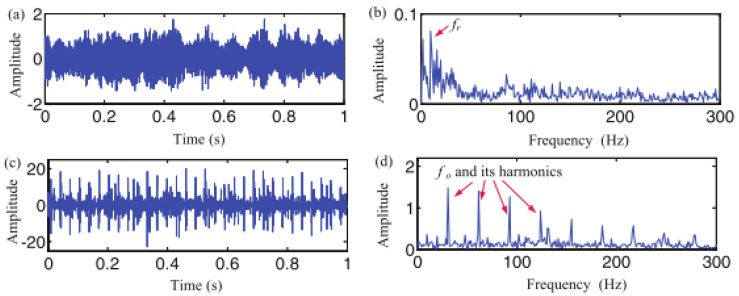
RSSD results and the instantaneous amplitude spectra of resonance components: (**a**,**c**) Optimal high and low-resonance components, respectively; (**b**,**d**) Instantaneous amplitude spectra of (**a**,**c**), respectively [52].

Soon afterward, Zhang et al. [52] developed a composite weight function as the objective function in GA and adaptively optimized *Q*-factors and redundancy factors synchronously. Equations (14)–(16) provide more details about the smoothness *SI* of high-resonance component and kurtosis *Kur* of low resonance component used in GA, as well as the weight function *F*:

(14) $$SI(x_{1})=\frac{\exp\left\{\sum_{i=1}^{N}\ln x_{1}(i)/N\right\}}{\sum_{i=1}^{N}x_{1}(i)/N}$$

(15) $$Kur(x_{2})=\frac{E\left\{\sum_{i=1}^{N}{\left(x_{2}(i)-\mu \right)}^{4}\right\}}{\sigma^{4}}$$

(16) $$F=\alpha_{1}\cdot SI(x_{1})+\beta_{2}\cdot Kur(x_{2})$$

where σ and μ denote the standard deviation and the average of original signal **x**, E{ } represents the calculation of the expected value, and $\alpha_{1}$ and $\beta_{2}$ are the weight coefficients of *SI* and *Kur*, respectively. This method was executed to process the signal collected from a gearbox fault test rig with a faulty gear and an outer race faulty bearing simultaneously. The optimized parameters based on the above-mentioned GA were *Q*_1_ = 4.25, *r*_1_ = 8.06, *Q*_2_ = 1, and *r*_2_ = 6.68. Figure 13 displays the resulting high- and low-resonance components and their instantaneous amplitude spectra. It can be easily seen that clear peaks occurred at the rotation rate *f_r_* (10 Hz) in instantaneous amplitude spectrum of the optimal high-resonance component, which indicated a gear fault. Similarly, the dominant peaks at outer race fault feature frequency, and its harmonics shown in Figure 13d, verified the existence of the outer race fault. The same method was also applied to diagnose the compound faults of a gear tooth crack and a bearing outer race fault.

Since the single *Q*-factor pairs can hardly represent all resonances of interference or wanted components, some researchers have attempted to solve this problem through the iterative algorithm. For example, Wang et al. [53] developed a comprehensive strategy based on RSSD and the iterative algorithm for the separation of full-oscillatory components from noisy machining vibration signals at the thin-walled parts machining process. When minimization of the *Q*-factor variation was reached, chatter-related low-resonance component and periodic cutting-related high-resonance component were separated successfully, providing a new means for monitoring the machining state. Comparing to Reference [53], in 2016, Shi [54,55] found that the oscillatory behaviors of high-frequency interferences in faulty rolling bearing vibration signals were manifold; thus a single high *Q*-factor failed to eliminate all interference signals at once. Inspired by this, an iterative RSSD was put forward to “peel” the interferences step by step, and rolling bearing fault feature information was successfully dug out from the final “purified” low-resonance component. It is important to note that even though the iterated objects are different, the basic point of these studies is identical. Specifically, it is the oscillatory property of one resonance component that is difficult to know beforehand, and a single *Q*-factor pair is highly likely to cause the loss of useful information. However, such an iterative RSSD will inevitably suffer from low efficiency, especially when dealing with large-scale data.

### 4.3. Subband Optimized RSSD in Mechanical Fault Diagnosis

**Figure 14:**
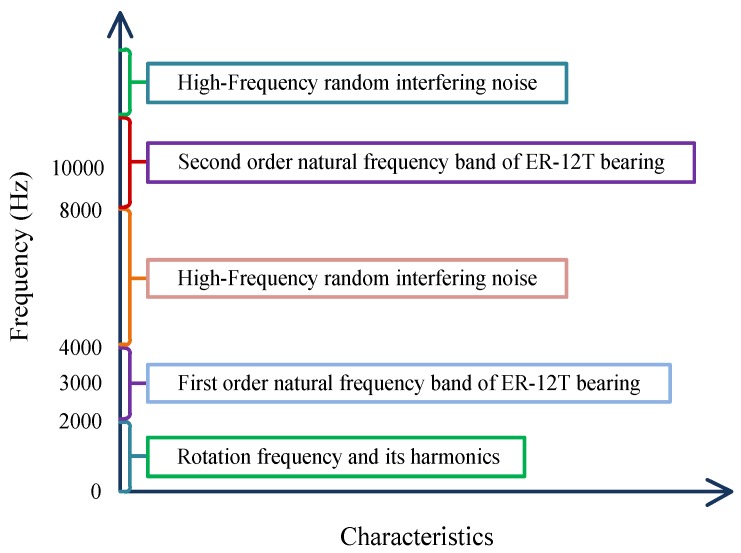
Frequency distribution of rolling bearing ER-12T.

**Figure 15:**
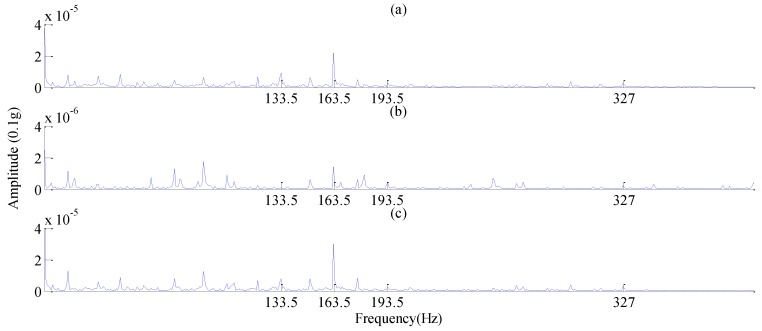
Main subband envelope spectra: (**a**) High-resonance component; (**b**) Low-resonance component; (**c**) Original signal [47].

Because there was still considerable noise in the resonance components obtained from RSSD, some scholars continually exploited the approaches for reconstruction of subband signals to further highlight fault features. Huang et al. [47] first proposed the concept of the “main subband”, and combined high- and low-resonance components to perform subband reconstruction in order to minimize the energy leakage mentioned in Section 4.1. As they described, for rolling bearing ER-12T, the rotation rate and its harmonic components were primarily concentrated below 2 kHz, with two natural frequency bands occurring at approximately 3 and 10 kHz, and the rest belonging to all kinds of interfering noise. Hence, the concrete frequency band distribution was gained, as viewed in Figure 14. Using Equations (8) and (9), the subbands approaching natural frequency bands, which were defined as the main subbands, can be calculated. After the main subbands of both high- and low-resonance components were obtained, the original signal’s main subbands can be obtained through superposing these two resonance components’ main subbands. The resulting main subband envelope spectra of the high-resonance component, low-resonance component, and original signal were illustrated in Figure 15a–c. As can be seen in this figure, although obvious peaks all occurred at the feature frequency (163.5 Hz), the corresponding amplitudes implied that the main subband of original signal brought the richest fault information, and thus it should be taken full advantage of, particularly when diagnosing incipient bearing faults.

Another subband reconstruction method was explored in Reference [56]. It merged the subbands of low-resonance component and selected the optimal signal with the biggest kurtosis for the extraction of fault features. Via this method, the fault features of a rolling bearing were prominent and easy to detect. As well as the direct mergence in Reference [56], Luo et al. [57] further combined these merged signals with similar kurtosis values and neighboring subbands. The final signal with the biggest kurtosis value was successfully used to diagnose a rolling bearing outer race fault. By selecting the components with extreme kurtosis values and removing those under the correlation coefficient threshold, Tang and Wang [58] successfully extracted weak characteristic frequency components of rolling bearings. Motivated by the neighboring coefficient thresholding for wavelet de-noising [59], He et al. [60] developed a neighboring coefficient de-noising (NCD) based RSSD. The subbands were processed through a neighboring coefficient thresholding scheme and then the reconstructed signal was obtained. The effective engineering applications, including bearing and gearbox fault diagnosis, validated its practicability and effect on suppressing noise. Given the sparsity and clustering/grouping property of subband coefficients [61], He and Zi [62] introduced a modified RSSD on the basis of overlapping group shrinkage (OGS) to facilitate the sparsity of results, and thus accomplished early fault diagnosis of rolling bearings.

### 4.4. Integrated Optimized RSSD in Mechanical Fault Diagnosis

**Figure 16:**
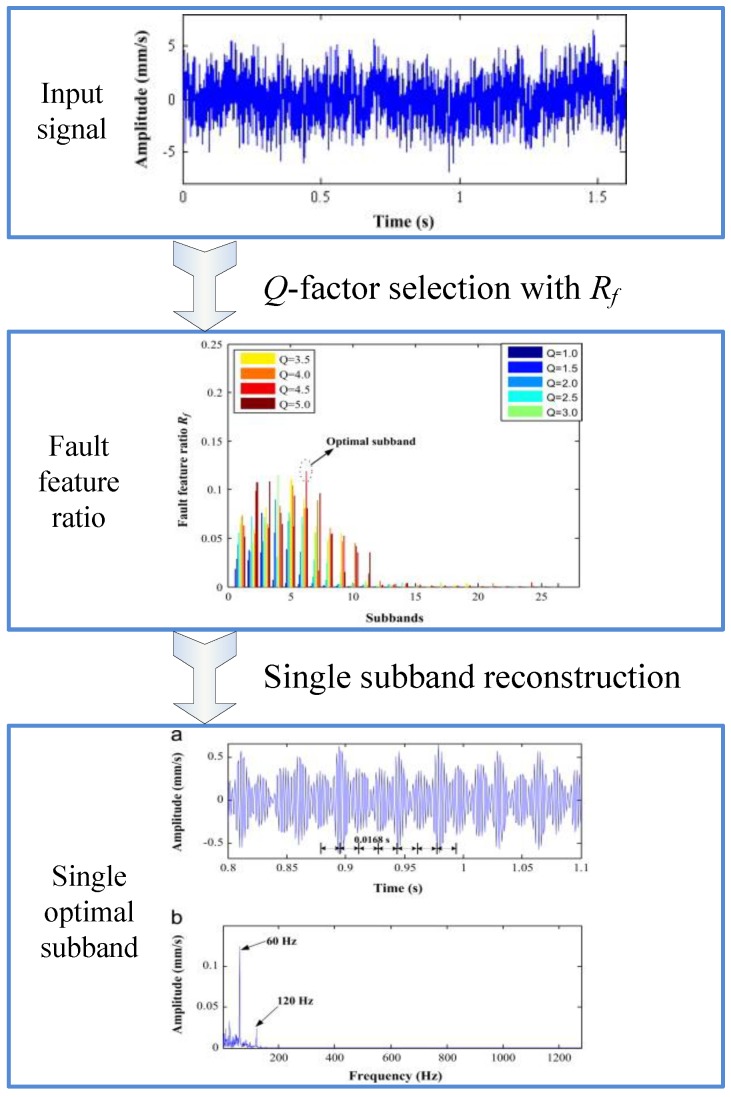
Example of bearing fault diagnosis via ensemble super-wavelet transformation (ESW) [63].

To pursue a perfect application of RSSD in mechanical fault diagnosis, numerous advanced RSSD techniques have recently been developed via combining parameter optimization and subband reconstruction methods, which show greater accuracy and adaptability in the field of mechanical fault diagnosis. An ensemble super-wavelet transformation (ESW) was proposed to investigate the vibration characteristics of bearing faults in temper mills and wind turbines [63]; the detailed flow chart is shown in Figure 16. With the fault feature ratio *R_f_* established by the Hilbert envelope spectrum, this method can adaptively select the optimal *Q*-factor. RSSD was subsequently performed on a faulty bearing’s vibration signal and the single optimal subband was extracted for subsequent reconstruction. As shown at the bottom of Figure 16, the impulses with period 0.0168 s (corresponding to 59.52 Hz) in the optimal subband time-domain waveform, and the obvious peaks at 60 Hz and 120 Hz in the frequency spectrum, all demonstrated that there was a defect on the outer race of bearing SKF-6232/C3.

In 2016, in response to the disadvantage in Reference [63] wherein the high-frequency interferences were not taken into consideration and the reconstruction of a single subband might lead to leakage of useful fault features, He et al. [64] improved the ensemble super-wavelet transformation. By modifying the definition of fault feature ratio *R_f_* and incorporating two optimal subbands, the modified method exhibited stronger robustness to high-frequency interferences and preserved more fault-related information. According to a priori information on the intrinsic periodic features of impulsive bearing faults, a periodic sparsity-based oriented super-wavelet transformation (PSOSW) was proposed [65]. An established weight index for the periodic sparsity feature energy ratio was adopted to guide the selection of RSSD parameters and reconstruction. The selected optimal super-wavelet bases were utilized for RSSD. This superior RSSD effectively discovered incipient weak fault features of a motor bearing installed on wind-power generation equipment. With the research on bearing fault mechanisms, Yu and Zhou [66] introduced an innovative method for diagnosing bearing faults using a combination of RSSD and spectral kurtosis. In accordance with the spectral kurtosis maximum principle, the optimal *Q*-factor and decomposition level were calculated. Meanwhile, the neighboring coefficient de-noising method was employed to eliminate the noise in the reconstructed signal, which resulted in a better application for bearing fault diagnosis.

### 4.5. RSSD Combined with Other Methods in Mechanical Fault Diagnosis

**Figure 17:**
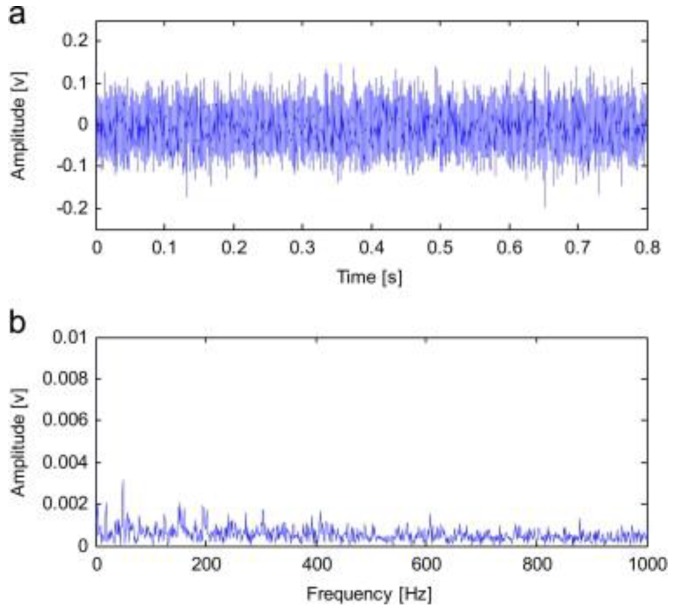
Rolling bearing early weak fault signal: (**a**) Time-domain waveforms; (**b**) Envelope demodulation spectrum [68].

**Figure 18:**
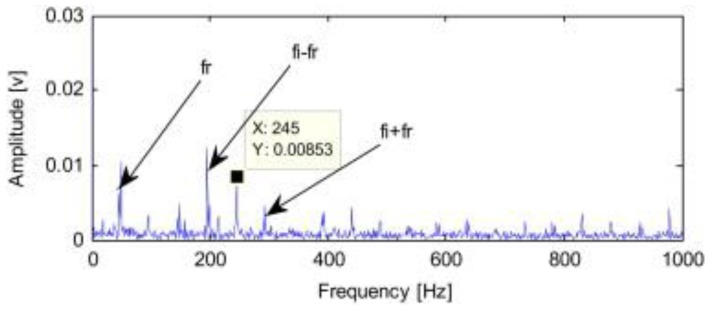
Envelope demodulation spectrum of IMF5 handled with RSSD [68].

This subsection reviews the applications of RSSD combined with other methods in mechanical fault diagnosis. To solve the problem that gear fault features are difficult to reveal under rotation rate fluctuations, Sun et al. [67] investigated an order-domain analysis based RSSD, which used the chirplet path pursuit algorithm to obtain rotation rate information. By performing order domain analysis on the low-resonance component obtained using RSSD along with the rotation rate information, the fault of gear with rotation rate fluctuation was identified. In 2014, Chen et al. [68] proposed an improved RSSD, based on ensemble empirical mode decomposition (EEMD) and TQWT, for the feature extraction of a rolling bearing’s early weak fault. Figure 17 and Figure 18 presented a typical example of bearing fault identification adopting their proposed method. The original signal of a bearing (type 6207) inner race fault was shown in Figure 17, including time- and frequency-domain signals where nothing fault-related can be readily observed. By applying EEMD, several intrinsic mode functions (IMF) were obtained. With a calculation with Equation (14), since IMF5 owned the biggest kurtosis, it was handled selectively using standard RSSD. The resulting envelope spectrum of the low-resonance component was shown in Figure 18. The peaks at the inner race fault feature frequency (246 Hz) and sideband frequencies manifested the effectiveness of Chen’s method.

Aiming at the ill-posed problem of blind source separation, Mo et al. [69] introduced a fast independent component analysis method (FastICA) on the basis of RSSD, which provided a new solution to the blind separation problem of the single-channel composite fault signal. The proposed method was then carried out to extract roller bearing composite faults. By integrating RSSD and fractional calculus, Yu et al. [70] analyzed the signal gathered by an oil particle sensor. With this method, the significant condition information of mechanical devices was provided in real time, owing to its advantageous computational efficiency and stability. To solve the sparsity problem required in compressed sensing (CS) and break through the limitation of signal length under the Shannon sampling theorem, a compressed fault-diagnosis method for roller bearings, based on RSSD, was proposed by Wang et al. in 2016 [71]. The application cases powerfully demonstrated that the bearing fault features could be extracted by the CS theory with RSSD even from the compressed samples. Combining RSSD analysis and manifold learning, an enhancement method was presented for periodic-impact fault features of rotating machinery [72]. Assisted by an impulse-enhanced signature index, it identified the rotating machine’s practical faults, including two defective bearing cases. Based on TQWT, Zhang et al. [73] established a kurtosis-based weighted sparse model utilizing two pieces of a priori information. By means of convex optimization, Zhang reliably extracted the outer race fault information of a deep-groove ball bearing using the estimated wavelet coefficients. In further research, Wang et al. [74] proposed an intelligent fault-diagnosis method for rolling bearings that made full use of TQWT, principle component analysis (PCA), and intelligent classifiers (nearest neighbor classifier and SVM classifier). The introduction of sparse wavelet energy (SWE) features greatly cinched this method’s success, including both fault feature extraction and pattern recognition of rolling bearings.

## 5. Summary and Discussion

**Figure 19:**
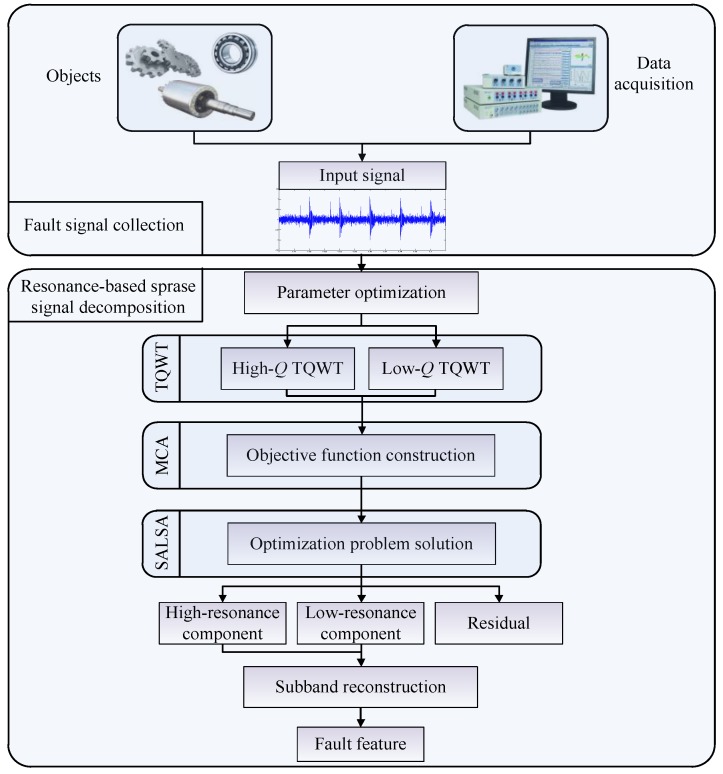
Flow chart of RSSD in mechanical fault diagnosis.

In Section 4, we have reviewed the applications of RSSD in mechanical fault diagnosis. In light of the numerous practical procedures and applications described, we believe that a clear and concrete flow chart is necessary, as shown in Figure 19. Note that both parameter optimization and subband reconstruction are included, so that such a flow chart describes a comprehensively optimized RSSD, which is of great value to guiding mechanical fault diagnosis. Meanwhile, due to the variety of approaches, the published research described above is also summarized in Table 4, for a one-page overview. The category, reference numbers, diagnostic objects, and supporting techniques are listed in the table.

From the application cases described in Section 4 and summarized in Table 4, the following tips are provided:

(1) At present, the applications of RSSD mainly focus on crucial industrial components like bearings, gearboxes, and rotors. Through RSSD, a mechanical fault signal is decomposed into the high-resonance component, low-resonance component, and residual, after which fault information can be extracted from these resonance components. In general, RSSD is considered as a powerful and excellent tool for the feature detection of mechanical faults.
(2) It must be stated that mechanical fault impact information will unavoidably scatter in high-resonance components, low-resonance components, and even residual components, due to the inherent coherence. This phenomenon can be thought as a kind of energy leakage. Different scholars are inclined to mine mechanical fault impact information from either high- or low-resonance components. Thankfully, they all have gained satisfactory diagnosis results. In fact, since these two perspective-based mechanical fault-diagnosis methods are both feasible, it is difficult to say that one approach will always outperform the others. Therefore, it is highly advisable to pay closer attention to the high- and low-resonance components simultaneously, as well as to the residual component. Moreover, it is the energy leakage that facilitates our seeking an optimized RSSD to preserve and extract mechanical fault-related information as richly as possible, which will be of great significance for early mechanical fault identification.
(3) To date, one feasible solution to reduce energy leakage and preserve the richest fault features is to optimize RSSD decomposition parameters, mainly including *Q*-factors and weight coefficients. With a view to these parameters’ significant effects on decomposition results, an artificially selected parameter pair may be not capable of discovering sufficient fault features. Even worse, ill-selected decomposition parameters might yield misleading diagnosis results. To avoid these problems and pursue satisfactory diagnosis, many techniques such as genetic algorithm, kurtosis index, and iterative algorithms, are successively introduced to adaptively acquire optimal *Q*-factors, weight coefficients, redundancy factors, and decomposition levels. Compared to original RSSD, parameter optimized RSSD can generate resonance components with richer fault impulsive information and less energy leakage, which will make great contributions to diagnostic decision-making.
(4) On the other hand, taking inevitable noise interference into consideration, fault information in resonance components obtained by parameter optimized RSSD may still be masked. As a result, many researchers have applied themselves to the energy distribution and reconstruction of subband signals, for the purpose of suppressing noise. Guided by a common concept, these studies have introduced specific indexes that can reveal fault information richness, and some de-noising techniques, including main subband, NCD, OGS, etc. The subbands with the richest information have been chosen to reconstruct mechanical fault vibration signals. It has been found that reconstructed signals with optimized subbands show outstanding performance in clearly detecting mechanical fault features, because broadband noise is obviously weakened. Moreover, an advanced RSSD, combining both parameter and subband optimization, will certainly perform better in diagnosing incipient mechanical faults than unilateral optimized RSSD, let alone original RSSD.
(5) In early stages of identifying mechanical fault types assisted with RSSD, researchers employed RSSD alone, or optimized it with decomposition parameters and/or subband reconstruction. Recently, many studies attempt to develop superior fault diagnosis approaches by combining standard RSSD with additional techniques, which are attracting more and more attention. For instance, after pre-processing by EEMD, the IMF with the biggest kurtosis value is analyzed for fault feature extraction using RSSD; CS theory is utilized to obtain the sparsest representation of resonance components from compressed samples, which breaks through the limitation of signal length. Successful applications prove that these specific RSSD-based methods can outperform standard RSSD in many applications and provide constructive guidance for diagnosing mechanical faults.

## 6. Outstanding Issues and Solutions

With RSSD’s swift advance, it has been proven fairly reliable in mechanical fault diagnosis. However, in addition to the above-described applications and developments, the following outstanding issues and solutions also deserve intensive discussion for advancing RSSD.

### 6.1. Construction of Wavelet Bases

As explained in Section 2, there are only two available methods to construct wavelet bases, RADWT and TQWT. Unfortunately, the wavelet waveforms constructed by both methods are symmetric in the time domain (see Figure 2). Due to machine operation, fault-induced vibration signals of mechanical components (represented by rolling bearings) usually exhibit distinct single-side damped oscillation characteristics after being transmitted through several mechanical interfaces. Hence, apparent inconsistency lies on the fault signal waveforms and wavelets obtained from RADWT or TQWT. Wang uses the single-side characteristic of the simulation signal to explain why the amplitude of resulting resonance component is halved [53]. However, taking the effects of decomposition parameters into consideration, especially the weight coefficients’ (see Figure 10 and Figure 11), conclusions drawn only from morphological characteristics aren’t convincing enough. Hence, when attempting to utilize a fixed method to construct wavelet bases with the aim of implementing all kinds of mechanical fault signal decomposition, wavelet bases will inevitably describe the morphological characteristics of fault signals inaccurately. To solve this problem, the following issues should be further investigated:

- Establish the dynamic models of mechanical systems and study vibration excitation mechanisms of various mechanical fault types; seek quantitative parameters that can characterize the morphology of fault vibration waveforms so as to reveal the mapping relationships between fault-induced vibration signal waveforms and mechanical fault types;
- On this basis, explore new construction means and construct targeted wavelet bases according to specific mechanical fault types, to make wavelet morphological characteristics match better with practical fault signals.

### 6.2. Parameter Optimization and Subband Reconstruction

Parameter optimization and subband reconstruction are two important topics of RSSD, as well as the fundamental assurance of RSSD’s successful application to mechanical fault diagnosis. State-of-the-art techniques primarily recur to the qualitative analysis, GA, and kurtosis index. However, qualitative analysis lacks a strong theoretical foundation, while objective function and prematurity in GA remain to be settled, and kurtosis is sensitive to noise interferences and unable to reflect the variation characteristics of fault impacts. All these shortcomings call for a more effective means in aspects of parameter optimization and subband reconstruction. Future work should pay close attention to the following aspects:

- Thoroughly study parameters’ influences on decomposition results, mainly including the influences of *Q*-factors and weight coefficients, then set up reasonable indexes to quantitatively evaluate their influence levels;
- Based on quantitative influence levels, study parameters’ coupling effects on RSSD results; establish a multi-parameter fusion optimization problem and take full advantage of multiple optimization ideas to realize the highly efficient optimization of decomposition parameters;
- Research the intrinsic links between fault signal features and subband energy distribution of resulting high- and low-resonance components; then put forward new methods for subband reconstruction to guide the core feature extraction of mechanical fault vibration signals.

### 6.3. Sparse Decomposition of Multiple Resonances

Aware of the multiple resonances existing in fault signals, the authors attempted to separate different resonance components via the iterative algorithm [53,54,55]. However, the iterative algorithm requires that resonance differences of the hidden components be rather distinct. Moreover, the resulting burden on time and memory, as well as the establishment of convergence indexes, are also quite challenging. Therefore, it must break through these bottlenecks and exploit effective sparse decomposition of multiple resonances when dealing with multiple signal components that own indistinctive resonance differences. To this end, three important directions are highly suggested:

- Investigate methods that can reduce inherent mutual coherence between resonance components, to promote the effective separation of each resonance component. As is known, mechanical fault-induced periodic signals usually correlate to natural frequency bands of systems, and the energy of resulting resonance components will merely concentrate on some specific subband groups. Considering that different signal components may lead to different frequency bands, a potential means is to utilize the specific subbands corresponding to respective frequency bands for signal decomposition with several resonances, rather than all *J*-level subbands. In this way, mutual coherence can be effectively dealt with. Additionally, as shown in Figure 4 and Figure 7, there is significant overlap of several adjacent subband responses: a latent scheme guides us to filter out some middle subbands whose frequency range can be filled up with their neighboring subbands. The vacant frequency ranges belonging to the filtered subbands can be prepared for another resonance component’s subbands. In this way, mutual incoherence between each resonance component can be feasibly enhanced. Notably, determining which subbands to filter relies heavily on the calculation of subband *f_c_* and *BW* with Equations (8) and (9), which needs a certain stock of a priori knowledge.
- Establish the optimization problem consisting of multiple resonances and develop a fast algorithm to seek its minimization;
- For potential mechanical fault types and resonances, seek characteristic variables or vectors that can describe mechanical fault vibration waveforms with several similar resonances. This direction offers an opportunity to further distinguish and separate resulting resonance components in view of signal structures.

## 7. Concluding Remarks

In this paper, we attempt to synthesize and review the theoretical developments of RSSD and its applications in mechanical fault diagnosis. As is known, RSSD is a new nonlinear signal separation method depending on resonance rather than frequency or time scale, which solves the frequency overlapping problem of each component and promotes the sparsity of resonance components. Consequently, it has become a new hotspot for numerous fields closely related to signal processing, especially the field of mechanical fault diagnosis.

So far, the main application objects of RSSD in mechanical fault diagnosis include bearings, gearboxes, and rotors, etc. In this review, based on the analysis of parameter effects and optimization directions, the application studies of RSSD have been classified into five categories: (1) original RSSD; (2) parameter optimized RSSD; (3) subband optimized RSSD; (4) integrated optimized RSSD; (5) RSSD combined with other methods. Despite preliminary achievements, there are some outstanding issues remaining to be discussed and solved, such as the construction of wavelet bases, suitable selection of parameters, subband reconstruction, and multi-resonance sparse decomposition. Specific approaches to these problems have been also pointed out.

In conclusion, the intention of this paper is to synthesize and review the scattered research on the developments and applications of RSSD in mechanical fault diagnosis. This review expectantly provides an in-depth and comprehensive reference for researchers who are concerned with RSSD and mechanical fault diagnosis. Moreover, we hope more advantageous RSSD will be developed and play an increasingly significant role in mechanical fault diagnosis.

## Figures and Tables

**Table 1:** Summary of parameters in TQWT.

Parameter	Symbol	Function Description
Quality factor	*Q*	Resonance degree
Redundancy factor	*R*	Overlapping rate between subband frequency responses
Decomposition level	*J*	Decomposition frequency range

**Table 2:** Correlation coefficients when *Q*_1_ varies from 2 to 9.

*Q*_1_	*Q*_2_	Correlation Coefficient	Correlation Coefficient
		(High)	(Low)
2	1	0.3067	0.9710
3	1	0.3072	0.9656
4	1	0.3072	0.9703
5	1	0.3068	0.9710
6	1	0.3065	0.9721
7	1	0.3063	0.9730
8	1	0.3063	0.9743
9	1	0.3059	0.9754

**Table 3:** Correlation coefficients in cases where $\lambda_{1}$ varies from 0.1 to 0.8.

$\lambda_{1}$	$\lambda_{2}$	Correlation Coefficient	Correlation Coefficient
		(High)	(Low)
0.1	0.1	0.9182	0.8351
0.2	0.1	0.3856	0.9622
0.3	0.1	0.3072	0.9703
0.4	0.1	0.3057	0.9745
0.5	0.1	0.3053	0.9792
0.6	0.1	0.3048	0.9838
0.7	0.1	0.3041	0.9882
0.8	0.1	0.3032	0.9920

**Table 4:** Summary of RSSD applications in mechanical fault diagnosis.

Category	Objects	Supporting Techniques	References
Original RSSD	Bearing	None	[32,34,35,36,37]
	Gearbox	None	[33,38,39,40]
	Rotor	None	[41,42,43]
	Others	None	[44]
Parameter optimized RSSD	Bearing	Qualitative analysis	[45,46,47]
		GA	[49,51]
		Iteration	[54,55]
	Gearbox	Evaluation index	[48]
		GA	[50,52]
	Others	Iteration	[53]
Subband optimized RSSD	Bearing	Main subband	[47]
		Kurtosis	[56,57,58]
		NCD	[60]
		OGS	[62]
	Gearbox	NCD	[60]
Integrated optimized RSSD	Bearing	ESW	[63]
		Improved ESW	[64]
		PSOSW	[65]
		Kurtosis, NCD	[66]
RSSD combined with others	Bearing	EEMD	[68]
		FastICA	[69]
		CS	[71]
		Manifold learning	[72]
		Kurtosis	[73]
		PCA, SWE, Classifier	[74]
	Gearbox	Chirplet path pursuit	[67]
	Oil	Fractional calculus	[70]
